# Timing Cellular Decision Making Under Noise via Cell–Cell Communication

**DOI:** 10.1371/journal.pone.0004872

**Published:** 2009-03-13

**Authors:** Aneta Koseska, Alexey Zaikin, Jürgen Kurths, Jordi García-Ojalvo

**Affiliations:** 1 Center for Dynamics of Complex Systems, University of Potsdam, Potsdam, Germany; 2 Departments of Mathematics and Institute for Women Health, University College London, London, United Kingdom; 3 Institute of Physics, Humboldt University Berlin, Berlin, Germany; 4 Potsdam Institute for Climate Impact Research, Potsdam, Germany; 5 Departament de Física i Enginyeria Nuclear, Universitat Politècnica de Catalunya, Terrassa, Spain; Center for Genomic Regulation, Spain

## Abstract

Many cellular processes require decision making mechanisms, which must act reliably even in the unavoidable presence of substantial amounts of noise. However, the multistable genetic switches that underlie most decision-making processes are dominated by fluctuations that can induce random jumps between alternative cellular states. Here we show, via theoretical modeling of a population of noise-driven bistable genetic switches, that reliable timing of decision-making processes can be accomplished for large enough population sizes, as long as cells are globally coupled by chemical means. In the light of these results, we conjecture that cell proliferation, in the presence of cell–cell communication, could provide a mechanism for reliable decision making in the presence of noise, by triggering cellular transitions only when the whole cell population reaches a certain size. In other words, the summation performed by the cell population would average out the noise and reduce its detrimental impact.

## Introduction

Genetically identical cells may exhibit diverse phenotypic states even under almost identical environmental conditions. An extreme example of this fact is provided by genetic switches, which can operate in one of two or more states that coexist. Such genetic switching is the basis of many cellular decision-making processes, including differentiation, whereby cells change their state when driven sufficiently beyond a certain threshold. A driving source for such processes might be in the form of environmental signals. However, switching can also occur cell-autonomously, when driven by stochastic fluctuations that unavoidably affect cellular behavior. In fact, noise is ubiquitous in gene expression [1], [2], [3], [4] and frequently cannot be neglected. Recent studies have indeed shown that sufficient amounts of noise are able to induce frequent jumps between coexisting states in genetic switches [5]. These results open up the question of how cells can make decisions reliably in the presence of noise.

Here we study the possibility that cell–cell coupling can provide a mechanism for enhancing the reliability of cellular decision making due to noise. Such a constructive role of coupling has already been discussed in the context of genetic oscillations in multicellular clocks [6], [7], [8]. In that case, precision enhancement arises from the synchronization of oscillations across the population, and is therefore associated with a homogeneous behavior of the cells. Here we discuss, on the other hand, a situation in which heterogeneity is preserved, but the decision making process is nevertheless reliable. A theoretical basis for these ideas has been established in general nonlinear stochastic models, where noise is known to be tunable through the size of the system, decreasing as the number of coupled elements increase [9]. Taking into account that cell populations increase their size autonomously (provided that sufficient nutrients are available and no growth-arrest signals are present), one can envisage a mechanism through which populations of cells self-organize into a minimum system size above which fluctuations are sufficiently small to allow a certain cellular behavior to arise. For population sizes below that critical value, commitment to a given cellular state would not take place due to the presence of an unacceptable amount of noise.

The mechanism outlined above requires a means of cell–cell communication in the growing cellular population. Eukaryotic cells, specially those forming part of multicellular organisms, have multiple ways to communicate; here we concentrate, for the sake of simplicity, on prokaryotic cells. Bacteria, for instance, have a mechanism of chemical communication [10] that relies on the exchange of small signaling molecules. These molecules, known as autoinducers (AI), freely diffuse through the cell membrane and are thereby shared by all cells in the population. Bacteria can thus use the external bath of AI molecules as a way of monitoring the density of cells in their surrounding. Such quorum sensing mechanism is used for instance by *Vibrio fischeri*, a bioluminiscent symbiotic bacterium that colonizes the light organs of certain types of fish and other marine species [11]. The *V. fischeri* LuxIR circuit has been used to build synthetic gene circuits, such as one performing programmed population control [12], and has been proposed as a method to obtain synchronization of genetic oscillators [7], [13].

Here we study how the interplay between noise, population growth and cell–cell coupling controls the dynamical behavior of a population of coupled genetic relaxators. These genetic circuits can exhibit bistable or oscillatory behavior when in isolation. Our results indicate that cell growth leads to reduction of noise (see also [14]) and appearance of clustering of the oscillators in the population, that can be interpreted as decision making. This mechanism works both when cells exhibit bistable or oscillatory behavior in the absence of noise, which evidences the generality of the phenomenon reported.

## Methods

### Structure of the model

**Figure 1:**
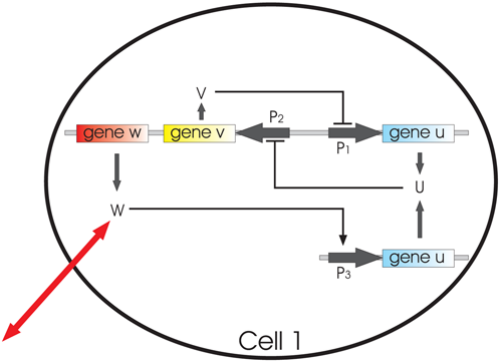
Simplified scheme of a genetic network in the frame of one cell. Mutually repressing genes *u* and *v* form a toggle switch. Membrane diffusion of an autoinducer molecule, denoted as *w*, provides intercell coupling.

We consider a model, proposed in Ref. [15] that describes a population of synthetic gene relaxator oscillators coupled via quorum sensing. The underlying genetic circuit (Fig. 1) contains a toggle switch composed of two genes *u* and *v* that inhibit each other, by repressing transcription from their respective promoters P_1_ and P_2_. This circuit is known to lead to bistable behavior [16]. Promoter P_2_ also drives the expression of a third gene *w* (corresponding to the *luxI* gene in the *V. fischeri* quorum sensing system) that synthesizes a small autoinducer molecule, which is able to diffuse in and out of the cell. The autoinducer activates transcription of promoter P_3_. Placing a second copy of the *u* gene under the control of this promoter provides both an additional feedback loop to the toggle switch, and a mechanism that couples the switch to all cells in the population via quorum sensing.

The time evolution of the proteins involved in the genetic circuit represented in Fig. 1 can be described by the following dimensionless equations:
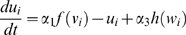
(1)

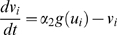
(2)

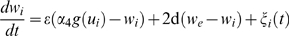
(3)

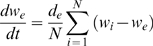
(4)where the subindex *i* denotes the cell number, with *N* being the total number of cells. The activity of the promoters *P_1_*, *P_2_* and *P_3_* described by the Hill functions *f(v)*, *g(u)* and *h(w)*, respectively, defined as:
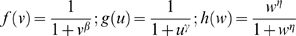
(5)


The parameters α_1_ and α_2_ determine the expression strength of the toggle switch genes, while α_3_ represents the activation of *u* from promoter *P_3_*. The expression of the lux gene *w* is measured by parameter α_4_. Time has been rescaled by the lifetime of *u* and *v*, assumed equal. The parameter ε measures the ratio between the lifetimes of the toggle-switch genes and the autoinducer, and is assumed to be small. This separates the dynamics of the cells into two very different time scales, with fast dynamics of *u*, *v* and *w_e_* and slow dynamics of *w*. The dynamics of the autoinducer (investigated in detail in [15]) introduces an additional feedback loop into the toggle switch and can lead to oscillatory behavior even in isolated cells [15]. The coupling coefficients *d* and *d_e_* depend mainly on the diffusion of the *AI* through the cell membrane. One can biologically manipulate the relevant parameters by controlling e.g. the number of plasmids per cell, protein decay rate or *pH* of the solution etc., which enables experimental control of the circuits dynamics. Stochasticity in gene expression is introduced in the autoinducer equation by an additive noise source ξ_i_ *(t)*, which is a Gaussian white noise with zero mean and correlation given by 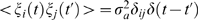. Adding the noise source to *u_i_* and *v_i_* leads to the same results as those shown in what follows (results not presented here).

## Results

### Controlling cellular decision making via population growth

**Figure 2:**
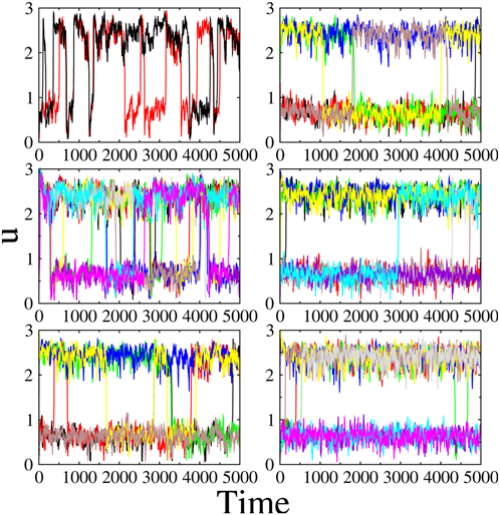
Time series for different number of cells with fixed noise intensity (*σ* *_a_^2^* = *0.002*). The dynamics of *u* is plotted for different cells in different colors. From top to bottom, and from left to right: *N* = *2*, *10*, *30*, *50*, *500*, *1000*. The parameters are chosen so that cells are in the bistable regime: α*_1_* = *2*, α*_2_* = *4*, α*_3_* = *2*, α*_4_* = *1*, *β* = *γ* = *3*, *η* = *1*, ε = *0.01*, *d* = *0.03* and *d_e_* = *1*.

First we analyze the situation in which the circuits operate in a bistable regime. This means that, in the unrealistic assumption that noise is not present, the concentrations of the observed proteins have one of two possible values. Noise, however, induces frequent jumps between the two stable concentration levels [5] and prevents the cell from making any stable decision between the two cellular states. Such a situation is shown in the upper left panel of Fig. 2 for two coupled cells.

We note that coupling in this system does not produce synchronization of the toggle switch dynamics because it acts incoherently with respect to it (compare the type of regulation of promoters *P_1_* and *P_3_*, the former being inhibitory and the latter activatory of *u* expression). This type of coupling is partially phase repulsive, promoting synchronization between two coupled elements only if they are close in phase space, and repulsion otherwise. Thus, inter-cell coupling does not have a dynamical effect on the bistable regime of the isolated cells. Its only influence reveals itself in an effective reduction in noise levels, similar to what has been reported in general models of nonlinear stochastic systems [9]. Figure 2 shows the effect of increasing the size of the population of coupled cells. As the system size increases, the amount of fluctuations in each cell is effectively reduced, which decreases the frequency of noise-induced jumps between both bistable states. For a large enough population size, all cells are stuck in one of the two states, and two stationary clusters of cells emerge. Only small percentage of the cells (*<2%*) exhibit rare noise induced jumps.

The previous results show that in a population of bistable switches under the influence of noise, robust decisions cannot be made unless the noise levels are reduced sufficiently so that fluctuations cannot induce jumps between both states, which can be accomplished by increasing the size of the cell population in the presence of cell–cell coupling. We can therefore envision a mechanism in which decisions are timed to occur only when the population reaches a critical size, below which noise is too large for a stable response to develop. We emphasize here that such a mechanism does not require a deterministic transition in the steady-state behavior of the system (something which quorum sensing can achieve), but only a control of the noise level via the system size. Therefore, the metabolic load in each cell would be comparable before and after the decision has been made.

**Figure 3:**
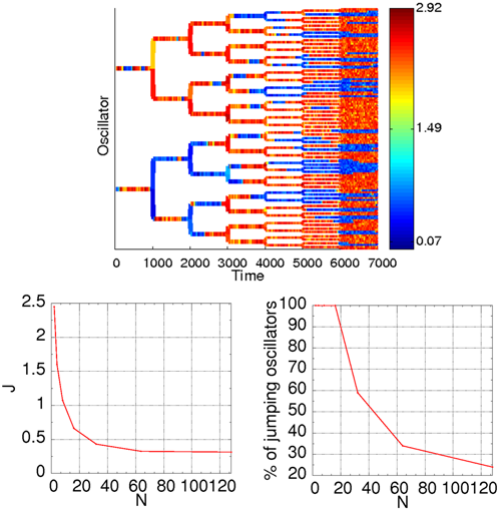
Top: population growth leads to restoration of bistability hidden by noise. The cell cycle duration is *T* = *100*. The concentration *u_i_* of the corresponding cell (from *N* = *2* to *128*) is color-coded (see color bar on the right). Bottom left: average number of jumps per cell versus population size. Bottom left: fraction of cells that perform at least one jump between two states versus population size. Parameters are α*_1_* = *2*, α*_2_* = *4*, α*_3_* = *2*, α*_4_* = *1*, ε = *0.01*, *d* = *0.03*, *d_e_* = *1* and *σ_a_^2^* = *0.002*.

In order to model this timing mechanism, we represent cell growth in a simplified way: after a given time period *T* all cells divide and the number of cells is doubled. All daughter cells start their dynamics with initial conditions for the protein concentrations equal to the final state of the mother cell. The behavior of this model is visualized in the top panel of Fig. 3 for a population of two initial cells, which grows until *N* = *128*. The concentration *u_i_* is plotted for every oscillator in color scale, with blue (red) representing a low (high) protein level. Initially the two cells exhibit noise-induced jumps (see also the top left panel in Fig. 2). As the cell population grows in size, the noise levels are reduced and the frequency of jumps also decreases, until all cells eventually get stuck in one of the two states. As a result, two approximately stationary cell clusters appear for a large enough size. This can be explained by the fact that effective noise in the system decreases with the population size as 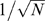.

To quantify how the decision-making dynamics changes as the population size increases, we define an order parameter, *J*, as the normalized number of jumps between the two stable states:
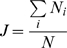
(6)where *N_i_* is the number of jumps above a certain threshold (here set to 1.5) for the *i*-th oscillator, in a given cell cycle, and *N* is the number of cells at that cell division round. A value of this order parameter approaching zero means that there exists either one or several stable clusters. The dependence of *J* on the population size *N* is shown in the bottom left panel of Fig. 3. The number of jumps per cell cycle for small *N* depends on the noise level; in our case the amplitudes of the fluctuations are such that each cell jumps more than twice between steady states every cell cycle. An increase in the population size (and, correspondingly, a decrease in the noise level) leads to a clearcut reduction of the occurrence of noise-induced jumps, reaching a plateau at *J*∼*0.3*, in which there is only approximately one jump on average in every three cell cycles. These results clearly show that cell growth leads to the restoration of bistability.

We can also compute the fraction of cells that jump at least once in each cell division round considered. This is depicted in the bottom right panel of Fig. 3, which shows that starting from a situation where two (out of two) cells jump from one state to the other, population growth reduces substantially the fraction of cells that jump up to a value around *25%* for *N* = *128* cells. No plateau is observed in this case; this shows that more and more cells get trapped in clusters of stable states for increasing *N*.

### Decision making in a population of coupled genetic oscillators

**Figure 4:**
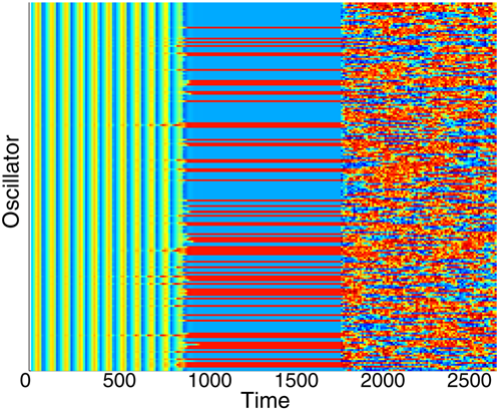
Effect of noise on a population of *N = 256* coupled cells in the oscillation death regime. The concentration *u_i_* is shown in color code according to the scale of Fig. 3. Time runs horizontally from left to right, while the different cells are plotted along the vertical axis, each cell represented by a horizontal line. Simulations were started with random initial conditions, and after a transient of around *900* time units (in which the oscillations are highly synchronous), two stable clusters emerge. At time *t = 1800* noise is switched on with intensity *σ_a_^2^ = 0.4*, and this leads to jumps between clusters. Parameters are *α_1_ = 3*, *α_2_ = 5*, *α_3_ = 1*, *α_4_ = 4*, ε = *0.05*, *d = 1* and *d_e_ = 30*.

We will now demonstrate that the phenomenon described in the previous paragraphs is a generic property of the interplay between noise and cell–cell communication. To that end, let us consider the case in which cells are originally (in the absence of noise) in an oscillatory regime (the *AI*, responsible for the coupling between the cells oscillates as well). Under these conditions, it is known [15] that coupling can suppress the oscillations via the mechanism known as *oscillation death*, leading to two clusters of cells with constant protein levels (when *OD* is achieved, the level of *AI* produced is also constant). Oscillation death is visualized in Fig. 4, where the dynamics of a fixed population of *N* = *256* cells is plotted in color code. Each cell is represented by a horizontal line, with color corresponding to its value of *u_i_*, using the same color scale as in Fig. 3. The plot shows that, in the absence of noise, oscillations develop from random initial conditions, and after some transient the cells get trapped in one of two possible states represented in either red or blue, forming two clusters. We note that coupling is here global, and thus these clusters do not have spatial order (without enough coupling, the cells undergo periodic oscillations that can be synchronized [15].). In a realistic situation, however, noise is present in the system. In the simulations shown in Fig. 4, noise is switched on at *t* = *1800*, and this causes all cells to jump randomly between the two states. Hence the clusters are destroyed by noise-induced oscillations.

**Figure 5:**
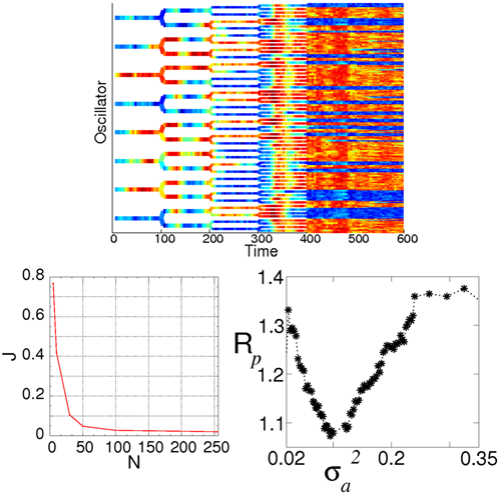
Top: population growth leads to restoration of bistability (in the form of oscillation death). The cell cycle duration is here *T* = *100*. The concentration *u_i_* of each cell (from *N* = *8* to *256*) is represented in color code (see color bar on Fig. 3). Bottom left: average number of jumps per cell versus population size. Bottom left: coefficient of variation of the interval between jumps versus noise intensity. Parameters are: α*_1_* = *3*, α*_2_* = *5*, α*_3_* = *1*, α*_4_* = *4*, *β* = *γ* = *η* = *2*, ε = *0.05*, *d* = *0.3* and *d_e_* = *1*.

As we have seen, oscillation death would allow decision making to occur even when the intrinsic dynamics of the cells is oscillatory. Noise, however, destroys this effect. On the other hand, in the light of the results presented in the previous section, we can expect inter-cell coupling to reduce the detrimental effect of noise and lead to robust decision making. In order to show this effect, we model again population growth by doubling the number of cells after a fixed cell cycle time *T*. The results are shown in Fig. 5, for a cell population starting with *8* oscillators operating in the oscillation death regime, and a noise intensity *σ_a_^2^* = *0.7* that for small population size leads to disordered jumps between clusters. As the population grows in size, the noise-induced oscillations become less frequent and eventually two clusters clearly develop for large enough number of cells (see Fig. 5, top). Again, the emergence of robust decision making as a result of population growth can be explained by the effective reduction of noise intensity as the system size increases. We note here that in the deterministic case for *N* cells, there are *N*-*1* possible stable different distributions of the oscillators between the two clusters [17]. Thus, the percentage of cells populating the upper or the lower cluster depends only on the environmental conditions (initial values, coupling coefficients etc.) and the system has no preference towards choosing ‘*u*’-(or ‘*v*’) rich cells. This statement holds true in both case, when the synthetic circuits operate in the bistable regime, as well as in the case where oscillation death is present in the system due to the global coupling present. Furthermore, we have investigated the stability of the achieved states by means of bifurcation and extended numerical analysis and shown that once a stable cluster distribution is achieved, the situation remains unchanged in latter times as long as the environmental conditions remain relatively stable (charts not shown here). Although both of the cases presented here lead to a stable decision making process by increase of the population size, it is important to mention that the noise levels tolerated by an oscillatory population are significantly higher than those of a system in a bistable regime. This contributes to the fact that increased connectivity in the network is accompanied by a more robust decision making mechanism.

Moreover, we quantify once again the restoration of bistability by computing the average number of jumps per cell and cell cycle. This is shown in the bottom left panel of Fig. 5. In this case, the parameters chosen are such that almost one jump occurs per cell and cell cycle ( *J*∼*0.8*) for a small population, while that fraction is reduced to around *2* jumps for every hundred cells (*J*∼*0.02*) for large enough population sizes (here on the order of *2^8^* = *256*). This result clearly indicates that noise-induced oscillations are prevented by an increase of the population size.

Interestingly, the top panel of Fig. 5 shows that for intermediate population sizes (here for *N* = *64*) the cells undergo synchronous oscillations. In order to understand this effect, we note that these cells have a well defined underlying time scale, determined by their oscillatory dynamics in the absence of coupling (also revealed in the transient dynamics of the noiseless system before clustering, see Fig. 4). The reduction of noise for increasing system size unveils the hidden clustering regime, but as a precursor of this a temporal synchronous behavior appears. This effect is a fingerprint of a phenomenon known as coherence resonance [18], or autonomous stochastic resonance, in which an optimal amount of noise enhances an intrinsic periodic behavior in stochastic nonlinear systems. In the present case the noise intensity, controlled by the system size, passes through this optimal value as the cell population grows, leading to synchronous jumps for an intermediate population size. To quantify this effect, we have estimated the regularity of the cellular dynamics by computing the coefficient of variation (normalized standard deviation) of the residence times in the two stable states, *t_p_*:
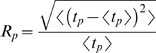
where 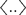 denotes time average. The bottom right panel of Fig. 5 shows how this quantity depends on the noise intensity for a fixed population of *N* = *8* cells. The figure shows that the regularity of the dynamics is maximum (the coefficient of variation is minimum) for an intermediate noise level.

Therefore, the emergence of synchronous oscillations in a population of coupled genetic circuits [7] can also be timed by the size of the population. In this way one can envision programming the start of a genetic clock only when a predefined population size is achieved.

## Discussion

The seeming paradox of how cells can operate reliably in presence of noise is being increasingly recognized recently. Specific gene-regulatory networks have been proposed to filter transcriptional noise so as to allow, e.g., coordinated developmental decisions to take place [19]. In this contribution we have proposed a mechanism that does not rely on any intrinsic property of single cells, but that emerges from the interaction among the cells (via small signaling molecules) in a growing population. The mechanism relies on an effective reduction of noise that occurs as the population increases in size. In such a way, a collection of bistable toggle switches that are continuously triggered by noise for small population sizes, would separate into clusters of cells stuck in one of the two coexisting states of the toggle switch for large enough population sizes, when the noise level is no longer sufficient to induce jumps between the two states. One could thus envision a mechanism for programming a decision to occur when the cell population becomes large enough: for smaller population sizes the cells would be undecided and jump randomly between two alternative states, whereas when the population grows to a sufficiently large size the cells would divide into two separate clusters, each one following an alternate fate.

Here we have assumed that the signaling autoinducer molecules diffuse very fast in the extracellular medium. Hence, coupling is global throughout the cell population and the resulting clusters do not reflect any spatial distribution. On the other hand, a limited diffusion range of the autoinducer would lead to a short-range, local coupling between the cells, which would in turn provide a patterning mechanism driven by the formation of spatial clusters. Programmed pattern formation driven by finite autoinducer diffusion has already been demonstrated in a synthetic gene-regulatory circuit in *E. coli* [20]. That mechanism, however, did not rely on a decision-making circuit.

Cell–cell communication has already been used to program a particular cellular process, namely cell death, in *E. coli* [12]. In that case, quorum sensing induces a transition between different dynamical regimes. The mechanism proposed here, on the other hand, does not rely on the occurrence of dynamical bifurcations, but only on the control of the intrinsic noise that is unavoidable in gene-regulatory networks. Noise reduction due to coupling has already been proposed as a mechanism of precision enhancement in multicellular genetic clocks [6], [7], [8]. That situation, however, relies on a homogeneous response of the system. The mechanism reported here, on the other hand, maintains the possibility that the system behaves in an heterogeneous way (something which is necessary in developmental processes, for instance), but nevertheless it is still able to benefit from the coupling-induced noise reduction.

A second effect of the intercell coupling discussed above, is the possibility that coupled genetic oscillators exhibit a phenomenon known as *oscillation death*. The presence of noise undermines the operation of the coupling-induced switch, in the same way that it prevents reliable decisions from taking place when the cells are intrinsically bistable but noisy. Again, decision making should in principle be possible for large enough population sizes.

Noise-reduction due to coupling has already been discussed in a biological context, mainly in the framework of neuronal dynamics. In that context, noise due to either (i) the random opening of ion channels [21], [22], (ii) fluctuations in the neurons' input currents [23], [24], and (iii) the incidence of a large number of stochastic synaptic inputs into a neuronal network [25] has been shown to be decreased with the system size. Here we propose, for the first time to our knowledge, a functional role for this effect at the level of gene regulation. As a prospect, it would be specially interesting to study how the growing diversity due to mutations would compete with the coupling-induced reduction of noise as the population grows.
